# 

**Published:** 1890-06

**Authors:** 


### LITERARY.

CHEAP DICTIONARIES A POOR INVESTMENT.—We feel it our duty to caution our readers against being deceived by purchasing by way of premium or otherwise, a cheap, by-gone and comparatively worthless issue of Webster's Dictionary, no nearer like the modern Webster, published solely by G. & C. Merriam, than a fossil is like the living form.

---

THE ANTHROPOLOGIST ; No. 1.—Published by the Anthropological Society of Boston, under the leadership of that well known student of the human brain, Prof. J. Rhodes Buchanan, has come to hand with assurances of future usefulness of which its membership is a sufficient guaranty.

---

VICKS' ILLUSTRATED MONTHLY.—Always good. We desire to express our acknowledgments to the publisher of the above excellent monthly for a generous supply of choice flower seeds.

---

Our thanks are due for the following treatises.

STRICTURE, ETC.—A Clinical Lecture, at N. Y. Post-Graduate M. S. Hospital ; by Prof. Charles D. Kelsey, M. D.

A NUMBER OF MEDICAL ESSAYS of value to the Profession ; by G. Frank Lydston, M. D., Chicago.

HEMORRHAGE AND ITS TREATMENT in certain cases ; by Thomas W. Kay, M. D., Scranton, Pa.

CARIES OF THE VERTEBRÆ AND ITS TREATMENT ; by Charles F. Stillman, M. D., Chicago.

SPLINTS FOR INFLAMED JOINTS, by same. Both illustrated.

THE SEWER GAS QUESTION.—By E. S. McClellan, M. D., New York.

MODERN CREMATION.—By le Dr. Prosper de Pietra Santa, Laureat de l'Institut (Académie des Sciences), Paris, France.

---

BABYLAND, makes its appearance for June. There is no other magazine published that is made especially for the babies—and by babies we mean the little ones from six months to six years of age. It will be found of great assistance to the mother in entertaining and amusing her baby. Only fifty cents a year. A specimen copy will be sent to any mother by the publishers, D. Lothrop Company, Boston.

TREATMENT OF TORTICOLLIS.—By Chas. F. Stillman, M. Sc., M. D., Chicago.

THE AUTOBIOGRAPHY OF GEORGE H. STUART.—This is a volume of 383 pp., interesting, not only as a truthful narrative of the events of a busy life, full of usefulness and honor, but as directly related to some of the leading charities organized for the amelioration of human suffering.

Mr. Stuart was President of the Christian Commission, which rendered so important a service in the field and elsewhere, during the period of the late "unpleasantness," and his autobiography furnishes the completest account of its work extant, and is otherwise historically valuable. In style, it is admirable, as being without any manifest attempt at rhetorical display, and we quite agree with the author, when urged as a representative of the commission, to confine its efforts more strictly to spiritual matters with less regard to the temporal, that "there is a good deal of religion in a warm shirt and a good beef steak."

The work is presented in excellent shape, with a number of illustrations, fac-simile letters and personal anecdotes of some of our most distinguished men with whom Mr. Stuart was brought into intimate relations.

Philadelphia, J. M. Stoddart & Co., publishers.

